# From genetic variation to precision medicine

**DOI:** 10.1017/pcm.2022.11

**Published:** 2023-01-24

**Authors:** Panagiotis I. Sergouniotis, Tomas Fitzgerald, Ewan Birney

**Affiliations:** 1 Division of Evolution, Infection and Genomics, School of Biological Sciences, Faculty of Biology, Medicine and Health, University of Manchester, Manchester, UK; 2 European Molecular Biology Laboratory, European Bioinformatics Institute (EMBL-EBI), Cambridge, UK; 3 Manchester Centre for Genomic Medicine, Saint Mary’s Hospital, Manchester University NHS Foundation Trust, Manchester, UK; 4 Manchester Royal Eye Hospital, Manchester University NHS Foundation Trust, Manchester, UK

**Keywords:** data integration, genetics, genomics, personalized medicine

## Abstract

Genetics has been an important tool for discovering new aspects of biology across life. In humans, there is growing momentum behind the application of this knowledge to drive innovation in clinical care, most notably through developments in precision medicine. Nowhere has the impact of genetics on clinical practice been more striking than in the field of rare disorders. For most of these conditions, individual disease susceptibility is influenced by DNA sequence variation in a single or a small number of genes. In contrast, most common disorders are multifactorial and are caused by a complex interplay of multiple genetic, environmental and stochastic factors. The longstanding division of human disease genetics into rare and common components has obscured the continuum of human traits and echoes aspects of the century-old debate between the Mendelian and biometric views of human genetics. In this article, we discuss the differences in data and concepts between rare and common disease genetics. Opportunities to unify these two areas are noted and the importance of adopting a holistic perspective that integrates diverse genetic and environmental factors is discussed.

Human health, well-being and behaviour are probabilistically shaped by the dynamic interplay between genetic and environmental factors. The landscape of genetic contributions to a given phenotype is referred to as its genetic architecture. This comprises the number of genetic variants that influence the phenotype; the magnitude of the variant effects; the variant frequencies in populations; and their interactions with one another and with the environment (Timpson et al., 2018; Benton et al., 2021; Visscher et al., 2021).

## Rare monogenic⟷common polygenic

**Figure 1.**
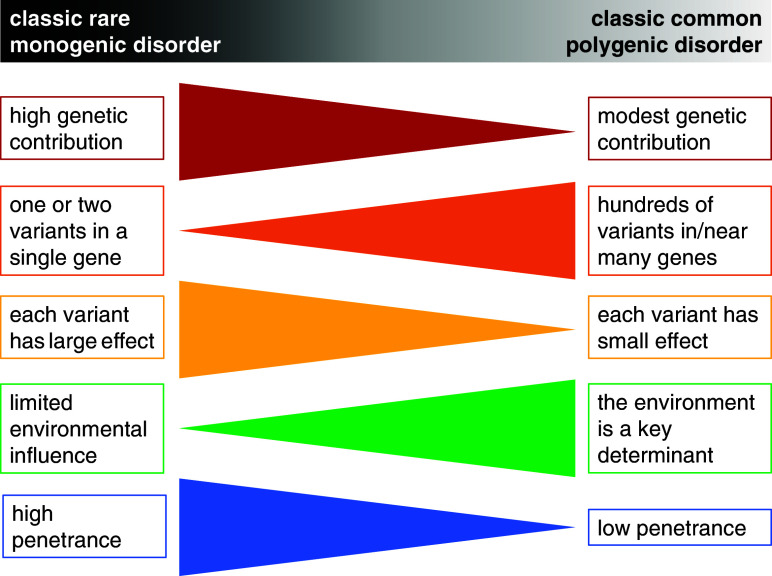
Key features of forms of human disease at the monogenic and polygenic ends of the genetic architecture spectrum. Notably, although the terms monogenic and polygenic formally refer to the number of genes involved in the genetic component of a disorder, they have come to mean broader styles of genetic inheritance anchored on the distribution of variant effect sizes (concept from Loos and Yeo, 2022).

**Figure 2.**
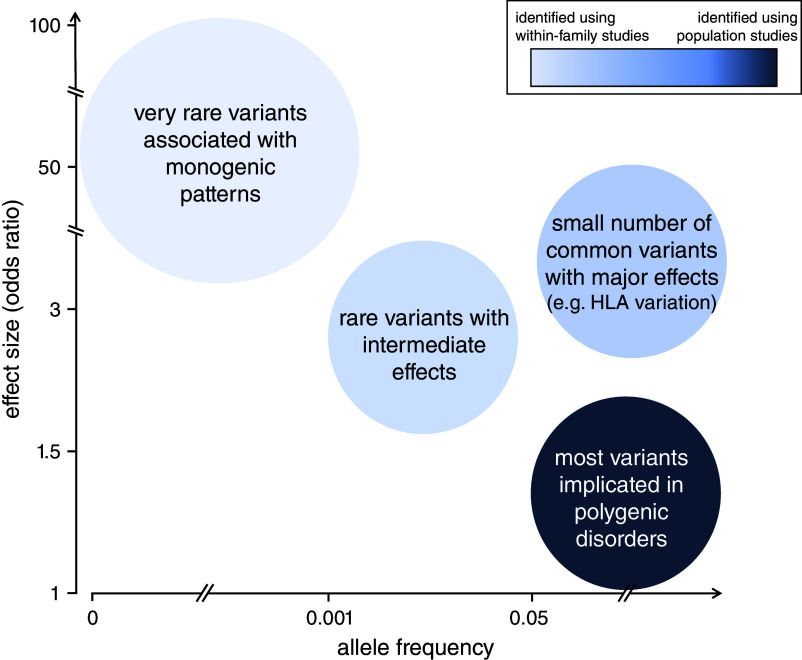
Schematic outlining the distribution of variant frequencies and effect sizes for key groups of genetic changes associated with human phenotypes. The minor allele frequency spectrum for these variants ranges from extremely rare to very common. In the context of conditions related to reproductive fitness, rare causal variants generally have larger effect sizes than common changes.

The terms ‘monogenic’, ‘oligogenic’ or ‘polygenic’ have been classically used to describe the genetic architecture of traits and disorders (Figure 1 and Table 1). The phenotypes at the monogenic (or Mendelian) end of the spectrum are rare and driven by a small number of low-frequency variants with large effects (Figures 1 and 2). Particularly relevant to these phenotypes are the concepts of recessiveness and/or dominance (which relate to the functional link between heterozygous genetic variants and the resulting phenotype). Mendel defined these concepts specifically for discrete, discontinuous traits without intermediate forms. He and others distinguished the characteristic inheritance patterns that bear his name (in which hybrids and one original strain have identical phenotypes) from additive patterns (in which hybrids have an intermediate appearance with noticeable contribution of both alleles to the phenotype) (Zschocke et al., 2022). It is worth noting that a significant proportion of the conditions described as dominant or recessive in the biomedical literature do not fulfil Mendel’s original criteria; many monogenic disorders, for example, exhibit semi-dominant or imperfect recessive inheritance with heterozygous carriers having a mild phenotype (Barton et al., 2022; Brandes et al., 2022; Zschocke et al., 2022).

**Table 1.** Selected examples of genetic architecture contexts

Genetic architecture context	Example	
	Disorder^1^	Genetic pattern
Extremely low-frequency variants with very large effect (including near-deterministic dominant disease related genetic changes)	Achondroplasia, a condition associated with altered bone growth and characteristic skeletal findings	Typically monogenic and fully penetrant; associated with heterozygous pathogenic (gain-of-function) variants in *FGFR3*
Low-frequency variants with a large effect that can be modified by other genetic factors	Autosomal dominant retinitis pigmentosa, a retinal condition associated with visual loss	Typically monogenic but clinically and genetically heterogeneous; heterozygous pathogenic (loss-of-function) variants in *PRPF31* are associated with incompletely penetrant forms (McLenachan et al., 2021)
Low-frequency variants with a moderately large effect that can be associated with digenic/oligogenic patterns	Left ventricular non-compaction, a congenital cardiomyopathy that can be associated with heart failure	Most cases are not monogenic; an oligogenic pattern involving variants in the *MKL2*, *MYH7* and *NKX2–5* genes has been described (Gifford et al., 2019)
Intermediate-frequency variants with a moderately large effect that can be associated with common, multifactorial disorders	Glaucoma, an optic neuropathy leading to a high risk of visual loss	Most cases are polygenic but heterozygosity for the *MYOC* c.1102C> T, (p.Gln368Ter) variant accounts for ~2% of cases (Zebardast et al., 2021)

1 It is noted that disease definition has an impact on the observed genetic architecture. For example, in certain disorders that are diagnosed after reproductive years (such as age-related macular degeneration [which can be associated with variants in the *CFH* gene] and Alzheimer’s disease [which can be associated with variants in the *APOE* gene]), large effect variants may lead to earlier and/or more severe clinical presentations.

The polygenic end of the genetic architecture spectrum includes a range of multifactorial conditions that are common and predominantly influenced by intermediate- and high-frequency variants across numerous genomic loci (each with a small effect size) (Figures 1 and 2; Claussnitzer et al., 2020). Genetic methods that can be used to study this group of conditions include genome-wide association studies (GWAS) and polygenic scores. These approaches assume additivity in the effects of genetic variants and generally have a ‘blind spot’ to phenomena like compound heterozygosity and recessiveness (Brandes et al., 2022). Empirical and theoretical evidence support this key additivity assumption, and linear (additive) genetic models appear to provide a sufficient approximation of the underlying biological complexity for many phenotypes (Hivert et al., 2021a,b; Brandes et al., 2022). It is however unclear if this picture emerges because of undue focus on a relatively narrow set of traits and disorders (and/or a requirement to use additive models for the discovery of genomic loci associated with these phenotypes).

**Figure 3.**
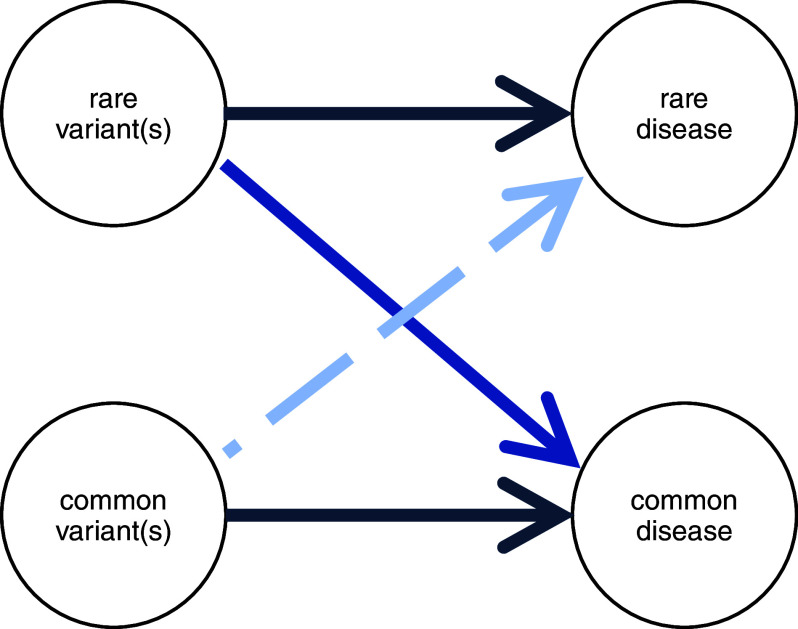
Challenging the ‘rare disease – rare variant’ and ‘common disease – common variant’ paradigms. The rare disease – rare variant hypothesis, predicts that if a disease with a significant genetic component is rare in the population, then the underlying genetic abnormalities will also be found to be rare. In the past decade, a number of studies have challenged this paradigm and have highlighted the role of common genetic variation in rare phenotypes (e.g., Niemi et al., 2018; Michaud et al., 2022). A related hypothesis has been made for common disorders; this proposed that if a disease with a significant genetic component is common in the population, then the genetic contributors will also be common. This common disease – common variant hypothesis has dominated the field for a number of years but has now been refuted; many examples of rare genetic changes contributing substantially to special cases of common disorders have now been described (e.g., Loos and Yeo, 2022).

**Figure 4.**
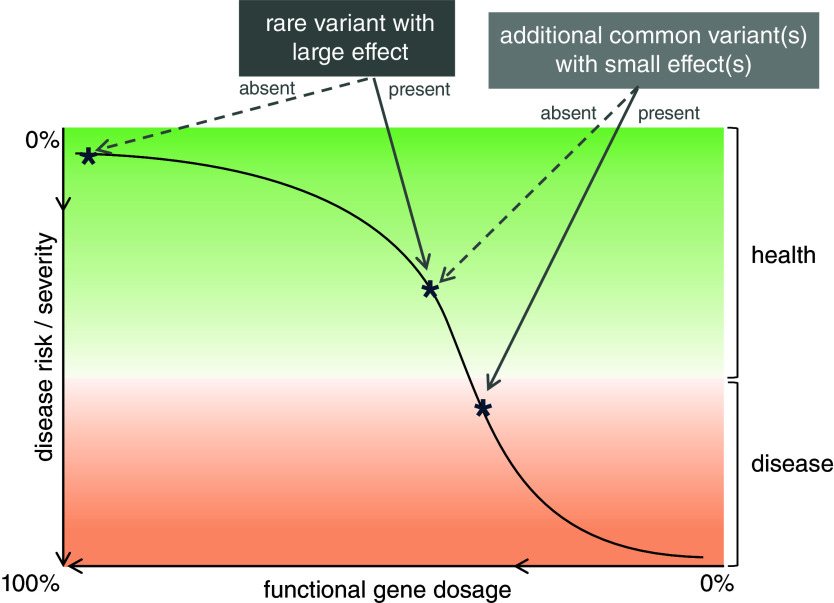
Schematic showing the joint effects of rare and common genetic variants on a disorder associated with a dosage-sensitive gene. In this hypothetical example, the presence of a rare variant results in loss-of-function of a copy of the affected gene, altering the background liability to the related disorder. This can be further modified by common variants with smaller effect sizes. In this case, the interaction between rare and common variation appears to push the individual beyond the disease threshold. It is noted that the variants may or may not interact in an additive fashion and that phase information is likely to be important.

It can be argued that dichotomising phenotypic spectra into rare monogenic forms (that are mediated by low-frequency variants) and common polygenic subtypes (that are mediated by high-frequency variants) is no longer productive and, to an extent, obstructs the discovery of new aspects of biology (Figures 3 and 4). In our work specifically on human eye development, we can see the convergence of the rare and common components of genetics. We have for example found that multifactorial traits like visual function and retinal structure are associated with the same high-frequency genetic variants that play a major role in albinism, a rare recessive condition (Currant et al., 2021; Michaud et al., 2022). We have also observed that combinations of common genetic changes in *TYR*, a major albinism-related gene that encodes the enzyme tyrosinase, can give rise to similar phenotypic manifestations to extremely rare loss-of-function variants in this gene. Notably, we have found evidence suggesting that the expressivity of loss-of-function alleles is altered by local and/or distal genetic interactions with other genetic changes (Michaud et al., 2022). Similar interactions between low- and high-frequency genetic variation have been reported in a number of rare and common phenotypes including Hirschsprung disease (Tilghman et al., 2019), Huntington disease (Lee et al., 2022) and blood cell indices (Astle et al., 2016).

## Family studies⟷population studies

The genetic architecture of traits and disorders can be studied using gene mapping approaches. During the 1980s and 1990s, efforts to map causal variants focused on rare monogenic phenotypes and mostly involved linkage studies in large pedigrees (Claussnitzer et al., 2020). In the 2000s, advances in genotyping array technologies (and the characterisation of the extensive linkage disequilibrium properties of human variation) enabled testing for associations between common phenotypes and genetic variation at a genome-wide scale. Early GWAS demonstrated the potential of these agnostic genomic surveys to highlight novel biological insights (e.g., *CFH* in age-related macular degeneration [Klein et al., 2005] or *IL23R* in inflammatory bowel disease [Duerr et al., 2006]), with the Wellcome Trust Case Control Consortium (https://www.wtccc.org.uk/) showing the broad applicability of these techniques (Claussnitzer et al., 2020; Crouch and Bodmer, 2020).

These successes have catalysed a shift from using family/pedigree data to studying whole populations at the genome-wide scale. More recently, however, there has been a renewed interest in conducting within-family studies (Uricchio, 2020; Visscher et al., 2021). These experimental designs are known to be efficient at dissecting ‘near monogenic’ phenotypes (including through the identification of *de novo* mutational events) but another key advantage is their ability to separate direct from indirect genetic effects. Indirect genetic effects include the influence of parental and sibling genotypes on the proband through alterations to the family environment (e.g., parents or older siblings can influence the school achievement or smoking behaviour of younger siblings) (Howe et al., 2022). Taking these indirectly causal factors into account is particularly important for understanding phenotypes with behavioural components (Kong et al., 2018). Overall, it is becoming increasingly evident that certain questions in human genetics are best answered using within-family studies and specially tailored experimental designs.

## Genotyping arrays⟷whole-genome sequencing

For the past two decades, genotyping of individuals participating in GWAS mainly involved using DNA arrays. These assays test a large number of intermediate- and high-frequency variants but generally overlook low-frequency changes, especially if these are in low linkage disequilibrium with neighbouring variants. Notably, it is now possible and increasingly cost-effective to comprehensively assay variation across the allele frequency spectrum using whole-genome sequencing. This approach is gradually replacing genotyping arrays as the method of choice for genetic association analyses (Uffelmann et al., 2022; Wainschtein et al., 2022).

A convergence has begun between what has been two distinct fields, one focusing on families and studying rare, monogenic phenotypes and one focusing on populations and analysing common traits and disorders. Methodological challenges remain (e.g., around addressing bias due to stratification or around incorporating phase information and structural variation) but large-scale sampling of families with whole-genome sequencing data is expected to help us build a more complete picture of the role of heritable variation in human phenotypes (Wainschtein et al., 2022; Young AI, 2022).

## Towards precision medicine

The drive behind studying the genetic architecture of human phenotypes follows a desire to explain and understand all the genetic contributions to human disorders. This knowledge directly informs the goals of medical genetics which include assisting in disease diagnostics and facilitating the identification of novel therapeutics. Furthermore, genetic studies are one of the building blocks of precision medicine which examines how an individual’s unique genetic and environmental/lifestyle characteristics come together to inform their health (Jameson and Longo, 2015; Ashley, 2016; Martschenko and Young, 2022). Below we provide a few examples of how genetic investigations can help us move away from ‘one-size-fits-all’ approaches to medical decisions and treatments.

First, genetic insights from gene mapping efforts can be used to obtain accurate molecular diagnoses. For many clinical presentations, there is great value in trying to refine the clinical diagnosis through genetic testing (which may involve DNA sequencing of disease-related genes, polygenic score estimation or a hybrid approach). The utility of genetic testing extends beyond rare phenotypes that are highly suggestive of a monogenic disorder (e.g., bilateral cataracts in a newborn). A notable clinical scenario is that of an individual with a common disorder (e.g., diabetes, obesity or cancer) who is found through genetic testing to carry a low-frequency genetic variant with a large effect. In a subset of cases, identifying such monogenic forms of common disorders can drive evidence-based changes in care management and result in improved outcomes (Loos and Yeo, 2022; Murray et al., 2022; Williams, 2022). It is worth noting however that, for most patients, obtaining a genetic diagnosis does not lead to a large therapeutic change. Nonetheless, an accurate diagnosis can improve planning and remove the need for inappropriate additional investigations which can be unpleasant and costly. Furthermore, it can have a big impact on affected families by providing a sense of closure/understanding or by allowing for better advice to be given regarding future reproductive choices. Overall, the use of diagnostic genetic testing in selected clinical presentations can make a difference to the affected individual (by better planning and sometimes better care), to their family (by providing closure and helping plan for other children if desired) and to the healthcare system (better planning, more targeted management).

Second, genetic discoveries can be used to develop tests that help identify subjects who are at a high risk of developing a specific disorder. Such predictive tests have been part of the care of families affected by certain monogenic conditions for a while, with non-invasive prenatal testing being a notable application (Zhong and Chiu, 2022). More recently, GWAS data have been used to create polygenic scores that aim to enhance disease risk prediction for common disorders (e.g., cardiovascular disease, glaucoma or breast cancer). The clinical utility of these tools for population-level screening will, to a large extent, depend on how they will be combined with other information including lifestyle factors, established biomarkers and/or the results of genetic tests that focus on low-frequency variant detection (Torkamani et al., 2018; Mars et al., 2020; Polygenic Risk Score Task Force of the International Common Disease Alliance, 2021; Szustakowski et al., 2021; Kullo et al., 2022).

Third, the identification of genetic variants contributing to human disease can inform therapeutic development and planning. Highly publicised examples of genotype-informed treatments include anti-PCSK9 cholesterol-lowering medications (Sabatine et al., 2017; Schwartz et al., 2018), BRAF/MEK-targeted therapy for metastatic melanoma (Vellano et al., 2022), triple-combination CFTR modulator therapy for cystic fibrosis (Middleton et al., 2019) and voretigene neparvovec intravitreal gene therapy for RPE65-related retinal dystrophy (Russell et al., 2017). These examples highlight that gene mapping studies can not only increase our understanding of the biology of human disease but also improve our practical ability to contribute meaningfully to their treatment.

## Inclusive genetics and the environment

The past 20 years have witnessed a rapid acceleration in our understanding of the genetic basis of many human disorders. With this greater understanding, it became possible to redefine disease at higher resolution and to target many disorders with precise therapies (Ashley, 2016).

In the near future, as whole-genome sequencing becomes the default assay, the artificial distinction between variants at the common and rare ends of the allele frequency spectrum will erode and it will become easier to consider the entire spectrum of genetic risk for an individual at once (McCarthy and Birney, 2021). However, the transition from array-based to sequence-based GWAS (McMahon et al., 2021) will require a sharper focus both on the development of appropriate methodology and on the collection of data from individuals/families with diverse ancestries.

**Figure 5.**
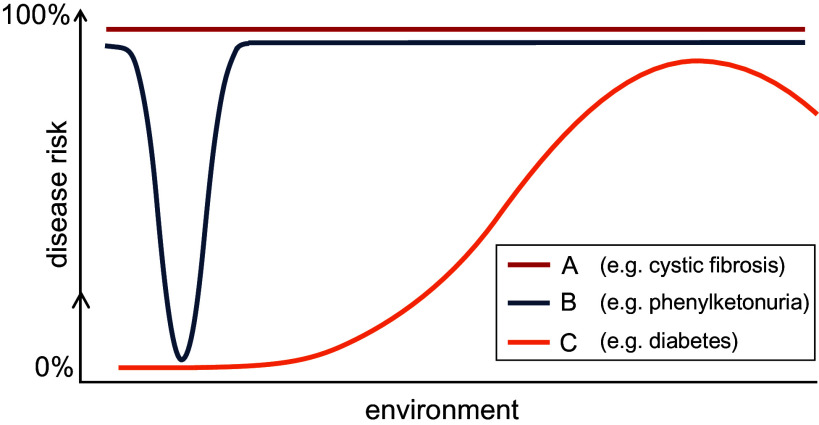
Schematic showing how genetic and environmental factors interact to produce human disease phenotypes. Disease can be defined as “a state of individual homeostatic abnormality (…); an aberration of adaptation in the face of conditions which are suboptimal, not necessarily for all, but for [at least] one genetically and socially distinct individual” (Childs, 1977). Hence, disease risk (*y*-axis) can be plotted as a function of genotype (coloured lines) and environment (a multidimensional parameter that is shown here for visualisation purposes as one dimension at the *x-*axis). Some genotypes are associated with high-penetrance monogenic phenotypes (such as cystic fibrosis) and lead to disease in all environments (line A). Other diseases occur only in the case of a very specific pairing of genotype and environment; phenylketonuria falls into this group as it manifests in individuals who carry biallelic loss-of-function *PAH* variants but only in the context of a diet that includes phenylalanine (line B). Most diseases fall between these extremes (e.g., diabetes; line C) and arise from ‘mismatches’ between genotype and environment (modified from Benton et al., 2021).

Genetic factors are one of the many aspects to consider when studying disease risk or contemplating precision medicine approaches. Environmental factors, a reductionist label referring to a range of non-genetic parameters, heavily influence most traits and disorders. Such factors include generic external exposures (e.g., social capital, education, financial status), specific external exposure (e.g., infectious agents, chemical pollutants, radiation), and internal exposures (e.g., metabolism, hormones, physical activity) (Peters et al., 2021; Canali and Leonelli, 2022). Another important parameter is time. Time and timing are critical to understanding how genes and environments operate together to shape probabilistically the trajectories of our lives (Figure 5). Experiences and exposures in early life for example are crucial elements of potential for success, failure, health or misfortune (Boyce et al., 2020).

Understanding the environmental contributors to specific disorders can highlight opportunities for treatment and prevention. It is known that, in certain scenaria, lifestyle changes can negate the development or progression of a disorder and may be as effective as any specific treatment; examples range from dietary interventions for rare inborn errors of metabolism such as galactosaemia to tailored lifestyle changes for chronic diseases such as hypertension and COPD (chronic obstructive pulmonary disease). To understand the role of targeted or broad interventions in various disorders and settings, the study of population-scale cohorts is required (as planned in the UK [Our Future Health], the USA [All of Us], Denmark, Iceland, Estonia, Finland and many other countries in Europe, Africa [H3Africa] and elsewhere). Additionally, there is a pressing need to improve the measurement and recording of environmental variables. Some of these factors can be imputed from the household location over a person’s lifetime and then cross-referenced to location-based environmental measures. However, many of the most important environmental parameters, such as the social environment around an individual, require individual measurement, ideally on a longitudinal basis. Here, the collaboration of geneticists with epidemiologists and sociologists will be critical, with each discipline bringing its insight into the holistic question of individual difference in phenotypes.

Ultimately, a deeper understanding of the interaction between genetic and non-genetic contributors to human disorders will allow a broader framing of disease risk, and will provide insights into how to develop optimal environments for each genetically unique individual.
