# Supplementary material for: From genetic variation to precision medicine
Source: Camb Prism Precis Med. 2023 Jan 24;1:e7. doi: 10.1017/pcm.2022.11 (PMC10953743; doi:10.1017/pcm.2022.11)
Supplement: Peer review history

# Reviewer report: Review: From genetic variation to precision medicine — R0/PR2

**DOI:** 10.1017/pcm.2022.11.pr2

**Authors:** Jennifer E. Posey

**Affiliations:** Baylor College of Medicine, United States

*Comments to Author*: In ‘From genetic variation to precision medicine’, the authors discuss the resolution of rare and common disease research and inclusion of environmental influence in the development of Precision Medicine. The authors provide a framework for conceptualize rare and common variation in human disease. I have several comments for the authors’ consideration.

Major comments

1. Oligogenic models of disease, including digenic conditions, dual molecular diagnoses, and mutational burden are not deeply addressed here and yet it seems critical that the authors include these more directly in the discussed models of disease resulting from rare and/or common variants and gene dosage effects.

2. One advantage of family-focused studies is the ability to identify de novo variants, and this should be addressed more directly within the text.

3. On line 288, where the authors list health-focused population cohorts, H3Africa and AllofUs might also be mentioned.

4. Figure 1: the colored triangles and their intended interpretation are somewhat unclear. The first three items (genetic contribution, # variants, effect size) seem to mirror the height of the triangle — I.e. effect size on the left (monogenic) is on the taller side of the triangle suggesting a higher effect size, whereas effect size on the right (polygenic) is at the tip of the triangle suggesting a smaller contribution. However, the final two items (penetrance, environmental influence) seem to be aligned in the opposite fashion, with ‘high penetrance’ and ‘the environment is a key determinant’ both at the tip of the triangle. Some revision of the wording, or explanation of what is meant by the triangles may be helpful here to avoid misunderstanding.

5. Figure 4 is a bit tough to interpret. In part, this may be because the y-axis seems to indicate increasing disease risk as one moves further up the graph, however the area of the graph itself that is indicated as having crossed the disease threshold is the lower part of the graph (orange/red). Likewise, functional gene dosage appears to increase along the x-axis from left to right, however, the example of rare + common variants is likely meant by the authors to indicate a reduction in gene dosage.

Minor comments

6. Line 186: I believe ‘anti-PCK9’ was intended as ‘anti-PCSK9’.

7. I appreciated the table provided at the end of the manuscript, but was unable to find where it was specifically referenced within the main text.

# Reviewer report: Review: From genetic variation to precision medicine — R0/PR3

**DOI:** 10.1017/pcm.2022.11.pr3

**Authors:** Samuel Wangler

**Affiliations:** Baylor College of Medicine, United States

*Comments to Author*: In this perspective a hopeful future for genetics research in envisioned. The future of genetics in which common and rare disease genetics has a synthesis and synergism is the anticipated and hoped for goal. This is a refreshing perspective in that often the two fields of common disease GWAS studies and medical genetics are studied, treated and analyzed by different groups of individuals. Separate sessions at major conferences house these Mendelian genetics versus complex traits. However biological realities often show dramatic overlap in the genes involved in both. There is a perception of competition between groups doing these two types of research but the opportunities for synergy is where the authors emphasize. The authors point to emerging data showing that conditions like Huntington’s disease have common variant modifiers and that key components of common disease can be revealed through rare disease loci.

The authors wish to knock down some of the perceived barriers between these analyses. In the section on rare monogenic to common polygenic the argument is successfully laid out that the dichotomy between monogenic and polygenic is too simplistic and their example of eye development is an excellent one. For the rare or Mendelian traits the authors point out that many conditions labeled autosomal dominant, are in fact semi-dominant, and while this is a common sloppiness of definition in medical genetics, it does not actually point to a spectrum between rare and common. The authors don’t directly distinguish a spectrum between specific gene variants that appear to be deterministic versus those that are probabilistic. Modifiers notwithstanding an individual with enough repeats within a certain polyglutamine tract in their genome will inevitably have Huntington’s disease with certainty and this is a unique feature of some of the more rare monogenic conditions. The authors appear to be glossing over that distinction when they say

“In the near future, as whole-genome sequencing becomes the default assay, the artificial distinction between variants at the common and rare ends of the allele 202 frequency spectrum will erode and it will become easier to consider the entire 203 spectrum of genetic risk for an individual at once.”

While the field should strive to become facile at both common and rare ends of a wide spectrum, some of the differences between common probabilistic loci and ultra-rare deterministic variants is not artificial. An individual with specific mutations in FGFR3 will have achondroplasia for example. A medical geneticist might send a test in a clinic and provide a diagnosis on the basis of that molecular result, and the whole concept of a “molecular diagnosis” in those cases is somewhat dependent on how deterministic the variant is. An individual with a risk locus for diabetes or HTN may or may not ultimately have HTN so such results represent more of a challenge for a direct molecular diagnosis. As the authors point out, precision medicine results could however be part of a more wholistic risk assessment that could widely benefit population health. For that matter an individual with a very rare familial mutation in Rb or p53 has a probability but not a certainty of cancer, so there are rare variants that are not deterministic and suggest a spectrum of variant effects. In any case don’t support the argument that this distinction is entirely artificial when they also discuss phenomena such as dominance which they say is a blind spot for common approaches but essential for understanding rare variants. It might help if the authors could discuss genes that constitute intermediates between common and rare or blur the line with intermediate frequency but relatively large effect size.

In Family studies to population studies the authors lay out an argument of a resurgence in family based studies that will help the field grasp both common and rare variants. It is encouraging that the authors discuss a convergence of two distinct fields. The discussions of precision medicine and inclusive genetics offer great insights and the positive view of the future of genetics in synthesizing the perspectives and assumptions from common and rare variant studies is quite encouraging. Overall this perspective offers the reader a helpful and very positive view for the prospect of genetics to adopt more unified theories and approaches.

# Editor report: Recommendation: From genetic variation to precision medicine — R0/PR4

**DOI:** 10.1017/pcm.2022.11.pr4

**Affiliations:** Bristol Medical School, University of Bristol, United Kingdom of Great Britain and Northern Ireland

*Comments to Author*: Dear Dr. Sergouniotis,

The manuscript was fully evaluated by two independent peer reviewers who have raised a number of concerns which we ask you to address in a revised manuscript. We also ask you to provide a detailed point-by-point response letter, highlighting your responses to the review comments and a description of the changes you have made to the manuscript.

# Editor report: Decision: From genetic variation to precision medicine — R0/PR5

**DOI:** 10.1017/pcm.2022.11.pr5

**Affiliations:** Institute of Cardiovascular & Medical Sciences, University of Glasgow College of Medical Veterinary and Life Sciences, United Kingdom of Great Britain and Northern Ireland

No accompanying comment.

# Reviewer report: Review: From genetic variation to precision medicine — R1/PR7

**DOI:** 10.1017/pcm.2022.11.pr7

**Authors:** Jennifer E. Posey

**Affiliations:** Baylor College of Medicine, United States

*Comments to Author*: The authors provide a throughful framework for how to conceptualize rare and common variation, and environmental impact, from a precision medicine lens.

The authors have responded thoroughly to reviewer feedback.

I do not have additional feedback to provide.

# Reviewer report: Review: From genetic variation to precision medicine — R1/PR8

**DOI:** 10.1017/pcm.2022.11.pr8

**Authors:** Samuel Wangler

**Affiliations:** Baylor College of Medicine, United States

*Comments to Author*: The authors have made a number of improvements and have addressed all the concerns of reviews.

# Editor report: Recommendation: From genetic variation to precision medicine — R1/PR9

**DOI:** 10.1017/pcm.2022.11.pr9

**Affiliations:** Bristol Medical School, University of Bristol, United Kingdom of Great Britain and Northern Ireland

*Comments to Author*: Both independent reviewers have affirmed that the authors have provided sufficient responses to their peer review comments and have addressed all concerns.

# Editor report: Decision: From genetic variation to precision medicine — R1/PR10

**DOI:** 10.1017/pcm.2022.11.pr10

**Affiliations:** Institute of Cardiovascular & Medical Sciences, University of Glasgow College of Medical Veterinary and Life Sciences, United Kingdom of Great Britain and Northern Ireland

No accompanying comment.
